# Superficial Burn Wound Healing with Intermittent Negative Pressure Wound Therapy Under Limited Access and Conventional Dressings

**Published:** 2016-09

**Authors:** Thittamaranahalli Muguregowda Honnegowda, Echalasara Govindarama Padmanabha Udupa, Pragna Rao, Pramod Kumar, Rekha Singh

**Affiliations:** 1 Department of Plastic Surgery and Burns, Kasturba Medical College, Manipal, Karnataka, India;; 2 Department of Biochemistry, Kasturba Medical College, Manipal, Karnataka, India;; 3 Department of Plastic Surgery and Burns, King Abdul Aziz Specialist Hospital, Sakaka, Al-Jouf, Saudia Arabia;; 4 Department of Pathology, Kasturba Medical College, Manipal, Karnataka, India

**Keywords:** Burn, Healing, Intermittent negative pressure, Limited access dressing, Conventional dressing

## Abstract

**BACKGROUND:**

Thermal injury is associated with several biochemical and histopathological alteration in tissue. Analysis of these objective parameters in research and clinical field are common to determine healing rate of burn wound. Negative pressure wound therapy has been achieved wide success in treating chronic wounds. This study determines superficial burn wound healing with intermittent negative pressure wound therapy under limited access and conventional dressings

**METHODS:**

A total 50 patients were randomised into two equal groups: limited access and conventional dressing groups. Selective biochemical parameters such as hydroxyproline, hexosamine, total protein, and antioxidants, malondialdhyde (MDA), wound surface pH, matrix metalloproteinase-2 (MMP-2), and nitric oxide (NO) were measured in the granulation tissue. Histopathologically, necrotic tissue, amount of inflammatory infiltrate, angiogenesis and extracellular matrix deposition (ECM) were studied to determine wound healing under intermittent negative pressure.

**RESULTS:**

Patients treated with limited access have shown significant increase in the mean hydroxyproline, hexosamine, total protein, reduced glutathione (GSH), glutathione peroxidase (GPx), and decrease in MDA, MMP-2, wound surface pH, and NO. Histopathologic study showed that there was a significant difference after 10 days of treatment between limited access vs conventional dressing group, Median (Q_1_, Q_3_)=3 (2, 4.25) vs 2 (1.75, 4).

**CONCLUSION:**

Limited access was shown to exert its beneficial effects on wound healing by increasing ground substance, antioxidants and reducing MMP-2 activity, MDA, NO and providing optimal pH, decreasing necrotic tissue, amount of inflammatory infiltrate, increasing ECM deposition and angiogenesis.

## INTRODUCTION

Patients with deep and extensive wounds of major burn with multiple injuries are difficult to treat.^1^ Although the pathophysiological mechanisms of tissue injury remain unclear, there is increasing evidence that oxidative stress have an important role in the development of multiorgan failure after thermal injury.^2^ In thermal trauma, tissues are subjected to ischemia.^3^ It was shown that oxidative stress due to burn injury initiate an inﬂammatory cascade that includes acute phase protein synthesis, upregulation of inﬂammatory adhesion molecules and pro-inﬂammatory cytokine, such as interleukin-1 (IL-1) and tumour necrosis factor-a (TNF-a).^4^

These activated inﬂammatory cascade causes local and systemic neutrophil sequestration, which is source of reactive oxygen species (ROS)^5^ and contribute to delay healing in burn wound.^6^ It has been shown that burn injury, associated with lipid peroxidation is an important cause of oxidative damage to cellular membranes, and eventually cell death.^7^ Increased plasma malondialdehyde (MDA) levels and concentrations of lipid peroxides in the systemic circulation have been described after burn trauma.^8^

Enzymatic antioxidants offer defense mechanism against ROS includes Superoxide dismutase (SOD), catalase, glutathione peroxidase and a glutathione (GSH) is one of the major constituents of cellular defense mechanisms against oxidative stress.^9^ Tissue ischemia after burn depletes intracellular antioxidant levels^10^ and induces apoptosis in fibroblasts during wound healing.^11^ It was demonstrated that after burn injury there was an increase in the total proteolytic activity in human burn skin with increased activity of MMP-2 and 9.^12^ Elevated levels of MMP-2 and 9 also have been reported in human burn wound fluid.^13^

The relationships between wound pH and healing of wounds was previously investigated and was found that weak acidic environments significantly inhibit protease activity and may potentially promote wound healing.^14^ Burn trauma produces a significant inflammatory response^15^ and up-regulation of the inducible nitric oxide synthatase (iNOS) leads to excess NO production, which contributes to post-burn cellular damage.^16^ Despite recent advances in the management of burn care, thermal trauma is difficult to manage.^17^

Negative pressure wound therapy (NPWT) is non-invasive adjuvant therapy to treat burn wounds. It is claimed to increase blood supply to the wound and to induce the formation of granulation tissue, causes rapid healing through stimulating of re-epithelialization, proliferation of endothelial, fibroblasts cells thereby angiogenesis and collagen reorganization, decreases bacterial load, eliminates exudate and slough, reduces oedema and swelling, and maintains an optimal moist environment for proper healing.^18^

The clinical evidence supporting the use of continuous NPWT on burn wounds has been based largely on clinician perception, case series, small cohort studies and weakly powered randomised trials that constitute a substantial number of publications but an overall low amount of evidence. Evidences are lacking on use of intermittent negative pressure dressing that is more economical and acceptable clinically than continuous NPWT.^19^ In this survey, a prospective randomized study was carried out to compare wounds treated with limited access dressing (cycle of 30 minutes suction and 3 ^1^/_2_ hours rest) and with conventional dressing biochemically and histologically to assess the rates of wound healing.

## MATERIALS AND METHODS

The study is prospective randomized clinical trial which was carried out in the Department of Plastic surgery and Burns, Kasturba Hospital, Manipal, India. Institutional Ethics committee of Kasturba Medical College and Hospital, Manipal University approved the study protocol and study was registered to Clinical Trials Registry India, (Government of India) - CTRI number: CTRI/2015/01/005419. Informed consent was obtained from all patients or their next of kin before inclusion into the study. 

**Fig. 1:**
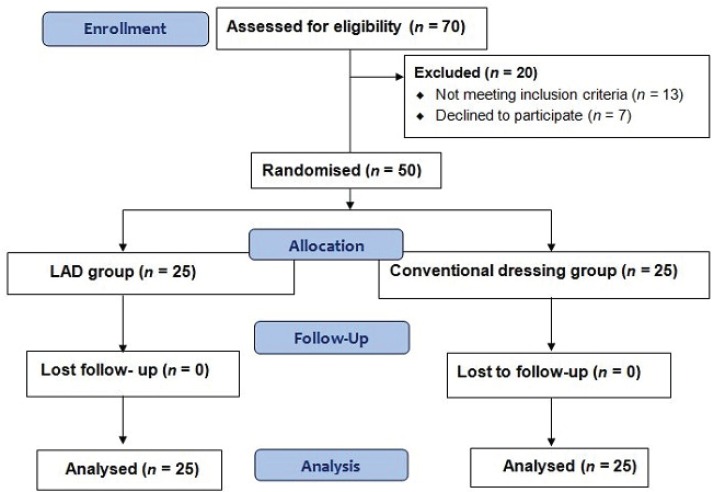
Consort flow chart.

Fifty patients of age 12 to 45 years (Mean age of 38.5 years) ailing from burn injury less than 40% total body surface area (TBSA) were enrolled in to the study. After examined inclusion criteria (less than 40% TBSA burn wounds) and exclusion criteria (Patients with collagen disorders, diabetic patients, leprosy patients, pregnant women, liver cirrhosis, HIV +ve ), they were randomly allocated to the limited access dressing (LAD) group (n=25), and conventional dressing group (n=25) (Figure 1) using simple randomization method, generating tables of random numbers through www.random.org. Numbers were assigned to a treatment group and sealed in opaque envelopes containing labelled paper with treatment and patient ID. Patient demographics and wound characterisation at baseline were shown in Table 1. On 0^th ^day biopsies taken from both groups, LAD group- patients were treated using LAD with intermittent negative pressure therapy. Conventional closed dressing group was treated with daily dressing changes using squeezed 5% povidoneiodine gauze which is a routine protocol in our burn unit.

**Table 1:** Patient demographics and wound characterisation at baseline

	**LAD group**	**Conventional group**
Number of patients	25	25
Age (Mean±SD) Age range (years)	32.3±9.50 12-45	34.5±13.0 17-43
Mean wound size (cm^2^)	19 (range=9-36)	18 (range=10-39)
Mechanism of injury (no.) Flames Scalds	23	22
	2	3
Female	9 (36.0 %)	10 (40.0 %)
Male	16 (64.0 %)	15 (60.0 %)

LAD: Limited access dressing; SD: Standard deviation

Standard L-Hydroxyproline, bovine serum albumin (BSA), standard glutathione, l-chloro-2,4-dinitrobenzene, nictoinamide adenine dinucleotide phosphate (reduced form), glutathione reductase (type III, Baker’s yeast), cumene hydrogen peroxide (Sigma–Aldrich, St. Louis, MO, USA), thiobarbituric acid, tri-chloroacetic acid, 1,1,3,3-tetramethoxypropane, N-ethylmaleimide (NEM), (SD Fine Chemicals Ltd, Boisar), MMP-2 Elisa kit (Uscn Life Science Inc. USA), pH paper strips, ortho phosphoric acid, (Merck, India), graded alcohol, Van Gieson stain (Sigma Aldrich Chemical Company, Bengaluru, India).

The granulation tissues were dried at 60°C for 24 hr. It was weighed and kept in glass stoppered test tubes. 6NHCl was added in each tube so that it contained 40 mg of the dried granulation tissue per ml of acid. The tubes were kept on boiling water bath for 24 h for hydrolysis. The hydrolysate was then cooled and excess of acid was neutralised by 10N NaOH using phenolphthalein/ Methyl red as an indicator. The volume of neutral hydrolysate was diluted to a concentration of 20 mg/ml of dried granulation tissue in the final hydrolysate with distilled water.^20^ The hydrolysate was used for the estimation of hydroxyproline and hexosamine

Granulation tissue samples wet weight was noted and homogenized by Rotex homogenizer in ice-cold 0.2 M phosphate buffer (pH=7.4). Homogenates were centrifuged at 15,000 rpm for 30 min in cooling centrifuge and supernatant was then used for determine total protein, antioxidants (GSH, GPx), oxidative biomarker (MDA). For MMP-2 assay, tissues we rinsed in ice-old PBS (0.1 mol/L, pH=7.0-7.2) to remove excess blood thoroughly and weighed. Minced the tissues to small pieces and homogenized them in 5-10 mL of PBS with a glass homogenizer on ice. The resulting suspension was sonicated with an ultrasonic cell disrupter or subjected to two freeze-thaw cycles to further break the cell membranes. After that, the homogenates were centrifugated for 5 minutes at 50×g, to remove the supernatant, aliquot and store at ≤-80^o^C.

For Nitric oxide (NO) assay tissue was homogenized in isotonic solution of PBS containing 10 mM N-ethylmaleimide (NEM) and 2.5 mM EDTA. The addition of NEM/EDTA served the purpose of blocking SH-groups and inhibiting transition metal-catalysed transnitrosation reactions, preventing artificial nitrosation, as well as thiolate and ascorbate-mediated degradation of endogenous RSNOs and nitrite. Protein concentration was determined according to Lowry method using purified bovine serum albumin as standard. Tissue hydrolysate was prepared and used for estimation of hydroxyproline,^20^ hexosamine,^21^ total protein,^22^ reduced glutathione (GSH),^23^ glutathione peroxidase (GPx),^24^ malondialdehyde (MDA),^25^ matrix metalloproteinase-2 (MMP-2),^26^ wound surface pH,^27^ and nitric oxide (NO).^28^

Wound biopsies on 0^th^ day and 10^th^ were collected, then fixed in 10% buffered formalin, dehydrated through graded alcohol series (50%, 70%, 90% and 100%), cleared in xylene and embedded (Leica EG1150 H) in paraffin wax (mp=56^0^C). Serial sections of 5-µm thickness were cut using microtome (Leica RM2255).The slides were stained with Van Gieson stain for the analyses. Each slide was given a histopathological score ranging from 1 to 12, with 1 corresponding to no healing and 12 corresponding to a completely reepithelialised wound.^29^ The scoring was based on the degree of cellular invasion, granulation tissue formation, vascularity, and reepithelialization. The histopathological score was assigned by investigator; code describing treatment to the patients was broken after the scoring was completed. 

Statistical analysis for biochemical parameters was performed by Student’s t-test and data were expressed as mean±standard deviation (SD). Histopathological score between the groups was performed by Mann-Whitney U test using the SPSS software (15^th^ version package, Chicago, IL, USA). The data were expressed as median and interquartile range (IQR). A *p* value <0.05 was considered as significant. When appropriate, statistical uncertainty was expressed by the 95% confidence levels. 

## RESULTS

Hydroxyproline (77.2±23.2 µg/mg dry tissue weight), hexosamine (9.4±2.8 µg/mg dry tissue weight), total protein (14.5±8.1 mg/g wet tissue weight), GSH levels (7.2±2.3 µg/mg protein), and activity of GPx (145.9±74.7 µmol/min/mg protein) were significantly higher in LAD group in comparison to the conventional group (29.7±14.8 µg/mg dry tissue weight, *p*=0.002 (9.1±1.96 µg/mg dry tissue weight, *p*=0.038), (9.21±4.2 mg/g wet tissue weight, *p*=0.013), (6.2±2.3 µg/mg tissue protein, *p*=0.039), and (115.9±68.8 µmol/min/mg tissue protein, *p*=0.001), respectively (Table 2).

**Table 2:** Results of biochemical parameters of LAD and conventional dressing groups

**Parameters**	**LAD Group (n=25) ** **[Mean±SD]**			**Conventional dressing group (n=25)** **[Mean±SD]**			***p*** ** value**
	**Day 0** ^th^	**Day 10** ^th^	**Day (0** ^th^ ** -10** ^th^ **)**	**Day 0** ^th^	**Day 10** ^th^	**Day (0** ^th^ ** -10** ^th^ **)**	
Hydroxyproline (µg/mg of dry weight of tissue)	62.1±12.6	139.7±25.7	77.2±23.2	67.5±10.4	100.5±18.0	32.7±14.8	0.002*
Hexosamine (µg/mg of dry weight of tissue)	6.79±1.5	15.7±2.5	9.4±2.8	7.0±1.4	15.4±2.5	9.1±1.96	0.038*
Total protein (mg/g of wet weight of tissue)	11.4±3.0	25.8±7.4	14.5±8.1	12.0±2.5	21.5±5.1	9.21±4.2	0.013*
GSH (µg/mg tissue protein)	15.9±4.3	22.9±3.3	7.2±2.3	14.9±3.5	21.2±3.5	6.2±2.3	0.039*
GPx (µMoles NADPH oxidized/min/mg tissue protein)	269.6±65.8	414.9±70.5	145.7±74.7	280±83.2	384.1±90.1	115.9±68.8	0.001*
MDA ( nmole/mg tissue protein)	20.1±4.45	6.79±2.24	13.6±6.62	19.7±4.17	9.45±4.0	9.86±3.35	0.006*
MMP-2 (ng/mg tissue protein)	0.96±0.80	0.66±0.42	0.54±0.45	0.77±0.52	0.56±0.49	0.41±0.34	0.024*
Wound surface pH	8.1±0.35	7.5±0.33	0.86±0.48	8.1±0.35	7.9±0.57	0.25±0.16	0.011*
Nitric oxide (µmole/mg tissue)	4.08±1.5	3.0±1.5	1.02±0.35	3.6±1.4	2.8±1.6	0.8±0.53	0.140

SD: Standard deviation; LAD: Limited access dressing; GSH: Reduced glutathione; GPx: Glutathione peroxidase; MDA: Malondialdhyde; NADPH: Nicotinamideadenine dinucleotide phosphate; MMP-2: Matrixmetalloproteinase-2

**Fig. 2:**
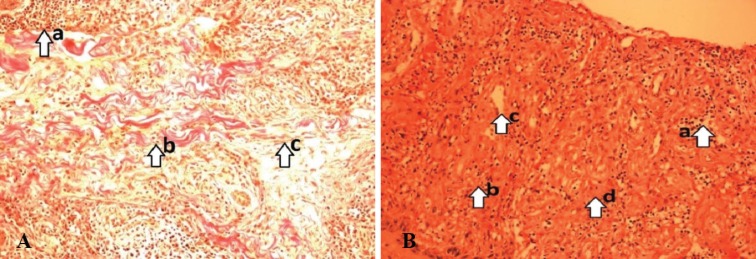
**A:** PreLAD (0^th^ day) (arrow) numerous neutrophils infiltration (a), minimum number of fibroblasts (b), fewer collagen fibers (c). (Photograph with Olympus PM20 photomicroscope 20X magnification). [VG stain]. **B:** PostLAD (10th day) (arrow) maximum number of fibroblasts(a), fewer inflammatory cells(b), More proliferating blood capillaries. (neovascularization) (c), Collagen bundles organized well between the cells (d). (Photograph with Olympus PM20 photomicroscope 20X magnification; VG stain

**Fig. 3:**
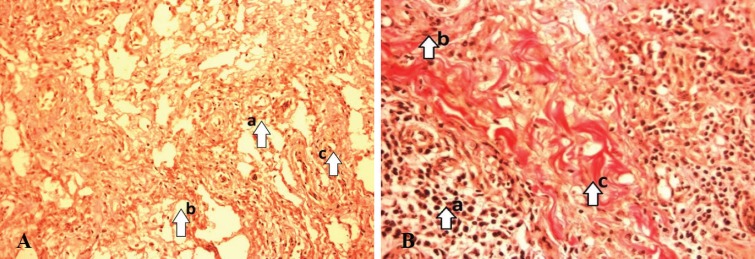
**A:** Pre conventional (0th day) - (arrow) numerous neutrophils infiltration (a), Minimum number of fibroblasts (b), poor collagen fibers (c). (Photograph with Olympus PM20 photomicroscope 20X magnification; VG stain). **B: **Post conventional (10th day) (arrow) numerous neutrophils infiltration (a), poorly developed matrix minimum number of fibroblasts (b), Poor collagen bundles (c). (Photograph with Olympus PM20 photomicroscope 20X magnification; VG stain

MDA was significantly higher in conventional dressing group (10.6±3.8 nmol/mg protein) when compared to the LAD group (6.5±2.24 nmol/mg protein, *p*=0.006). A significant decrease was noticed in the level of MMP-2 (0.54±0.45 ng/mg protein), wound surface pH (0.86±0.48), NO (1.02±0.35) when compared to the conventional group (0.41±0.34 ng/mg protein, *p*=0.024), (0.16±0.25, *p*=0.011), and (0.8±0.53, *p*=0.140), respectively (Table 3). On day 0^th^, both the LAD group [Figure 2A (VG)] and conventional group showed a necrotic tissue with increased cellular infiltration [Figure 3A (VG)]. On day 10, LAD group showed an increase in extracellular matrix (ECM) deposition, decrease in cellular infiltration and increased angiogenesis [Figure 2B (VG)] when compared to conventional dressing group [Figure 3B (VG)].

**Table 3:** Results of histological study of LAD and conventional dressing groups

**Group **	**N**	**Day 0 ** **Median** **(Q**_1_ **, Q**_3_ **)**	**Day 10** **Median ** **(Q**_1_ **, Q**_3_ **)**	**Day (0-10) Median ** **(Q**_1_ **, Q**_3_ **)**	**Wilcoxon Mann Whitney U test **
					***p *** **value**
LAD	25	5 (3 , 7)	9 (7, 9)	3 (2, 4.25)	0.008
Conventional dressing group	25	4 (3, 6)	6 (5, 7.25)	2 (1.75, 4)	

A Mann Whitney U test was performed to determine the significant difference in the average (Median) histological score of LAD and conventional group. The result indicates that there was a significant difference in the average score of the two groups (*p*=0.008). Median (Q_1_, Q_3_): Median interquartile range; LAD: Limited access dressing

On day 0, histological score in LAD versus conventional dressing group was 5 (Q_1_, Q_3_) (3, 7) and 4 (3, 6), respectively. On day 10, histological score in LAD versus conventional dressing group was 9 (Q_1_, Q_3_) (7, 9) versus 6 (5, 7.25). The test was performed for the difference of scores of day 10 and day 0 (LAD versus conventional dressing group) which was 3 (Q_1_, Q_3_) (2, 4.25) versus 2 (1.75, 4) (*p*=0.008). Consistent with these findings, the histopathologic score of wounds from LAD group was significantly higher with a decreased cellular filtration and increased fibroblasts, collagen deposition, increased the number of capillary vessels per high power field than conventional dressing group.

## DISCUSSION

Thermal burn initiates systemic inflammatory reactions producing burn toxins, oxygen radicals and finally leads to peroxidation. The relationship between the amount of products of oxidative metabolism and natural scavengers of free radicals determines the outcome of tissue damage.^30^ This can be determined by assessment of antioxidants (GSH, GPx). The study on lipid peroxidation product, the malondialdehyde levels gives us the extent of free radical damage in the cells.^31^

The formation of well-vascularized granulation tissue in the wound bed is a prerequisite for burn wound healing.^32^ Granulation tissue composed of fibroblasts, collagen, edema, and new small blood vessels. Extracellular matrix of granulation tissue contains most dominantly collagen which assists the wound and plays an important role in homeostasis.^33^ Collagen not only confers strength and integrity to the tissue matrix but also plays an important role in homeostasis and epithelialization in wound healing. Collagen is composed of the amino acid, hydroxyproline, has been used as a biochemical marker for tissue collagen.^34^

Various studies on human wound models shown that dressing technique like moist wound dressing^35^ and continuous NPWT^36^ increased the level of hydroxyproline content in wound granulation tissue. In the present study, the mean±SD hydroxyproline content in LAD group was 77.2±23.2 µg/mg of dry weight of tissue which was significantly higher than conventional dressing group (29.7±14.8, *p*=0.002). Hexosamine is an important part of the extracellular matrix and one of the main glycosaminoglycan secreted during tissue repair. Increased hexosamine content reflects the stabilization of collagen molecules by enhancing electrostatic and ionic interactions with it, which in turn reflects remodeling of the new extracellular matrix produced.^37^ Hence, enhanced hydroxyproline and hexosamine synthesis provides strength to repaired tissue and stimulates healing. In the present study, the mean hexosamine (±SD) in LAD vs conventional dressing group was 9.4±2.8 vs 9.1±1.96 (*p=*0.038).

Protein is another important constituent of extracellular matrix. High protein content confirms positive effects towards cellular growth and proliferation, granulation tissue formation and epithelisation.^38^ In the present study, mean±SD total protein was significantly higher in LAD group in comparison to the conventional dressing group (14.5±8.1 Vs 9.21±4.2 mg/g wet tissue weight; *p*=0.013). GSH (tripeptide) and enzymatic antioxidants which are normally present at high concentrations in intracellular, which constitutes the major reducing capacity of the cytoplasm and protects the cellular system against the toxic effects of lipid peroxidation.^39^ In the present study, LAD vs conventional dressing group for mean±SD GSH was 7.2±2.3 vs 6.2±2.3 (*p=*0.039), and for GPx was 145.7±74.7 vs 115.9±68.8 (*p=*0.001). MDA is produced during the attack of free radicals to membrane lipoproteins and polyunsaturated fatty acids.^40^ In the present study, MDA significantly decreased in LAD group (10.6±3.8 nmole/mg protein) compared to conventional group (6.5±2.24 nmol/mg protein, *p*=0.006).

Burn injury leads to an increase in the total proteolytic activity in human burn skin. MMP-2 has ability to proteolytically degrade type I, IV and V collagens, elastin and vitronectin, induces apoptosis in endothelial cells and inhibits neovascularisation.^41^ Several studies have shown in chronic wounds, including burn wounds, elevated expression and activation of MMPs -2 and 9, which may result in chronic tissue turnover and failed wound closure. In our study, LAD vs conventional group mean±SD for MMP-2 was 0.54±0.45 vs 0.41±0.34 (*p=*0.024).

The pH environment of chronic wounds has been recorded within the range of 7.15–8.9.^42^ This variability is representative of both healing and non-healing wounds. Both acute and chronic wounds with an elevated alkaline pH have demonstrated lower rates of healing than wounds in which the pH is closer to neutral.^42^ Consistent with these results in the present study mean±SD of wound surface pH (0-10^th^ day) LAD vs conventional group on the 0^th^ day was 0.86±0.48 vs 0.16±0.25 (*p=*0.011] and on the 10^th^ day was 7.5±0.33 vs 7.9±0.57.

Burn injury was associated with increased inflammatory response confirmed by increased serum nitric oxide levels. Overproduction of NO has harmful effects on vascular regulation.^43^ NO also interacts with the superoxide radical to yield peroxynitrite and other RNS, which are highly reactive. Peroxynitrite produces cellular death by enhancing DNA single strand breakage, cellular energy depletion and cellular necrosis.^44^ Consistent with above results in our study, NO mean±SD in LAD vs conventional dressing group was 1.02±0.35 vs 0.8±0.53 (*p=*0.140).

Various studies have shown that NPWT increase in rate of granulation tissue formation, angiogenesis, and decreased inflammation.^45^ Wound healing was markedly improved in LAD group (8.70±0.21) after 10 days of treatment when compared to the conventional dressing group (6.24±0.40). The Median (Q_1_, Q_3_) on 0^th ^–10^th^ day (LAD vs conventional dressing group was 3 (2, 4.25) vs 2 (1.75, 4; *p*=0.008). 

LAD exerts its beneficial effects on burn wound healing by significantally increasing ground substance, antioxidants and reducing oxidative stress, MMP-2, wound surface pH, and NO. An increase in ECM deposition and angiogenesis and decrease in necrotic tissue, and amount of inflammatory infiltrate were noted when compared to the conventional dressing group. Results of our biochemical and histopathological studies supports the clinical observation indicating better clinical effect of LAD on granulation tissue formation.

## CONFLICT OF INTEREST

The authors declare no conflict of interest.
